# Assessing the validity of the Global Activity Limitation Indicator in fourteen European countries

**DOI:** 10.1186/1471-2288-15-1

**Published:** 2015-01-02

**Authors:** Nicolas Berger, Herman Van Oyen, Emmanuelle Cambois, Tony Fouweather, Carol Jagger, Wilma Nusselder, Jean-Marie Robine

**Affiliations:** Public Health and Surveillance, Scientific Institute of Public Health, Rue Juliette Wytsmanstraat 14, 1050 Brussels, Belgium; Faculty of Public Health & Policy, London School of Hygiene & Tropical Medicine, London, UK; French Institute for Demographic Studies (INED), Paris, France; Institute of Health & Society, Newcastle University, Newcastle upon Tyne, UK; Department of Public Health, Erasmus Medical Center Rotterdam, Rotterdam, The Netherlands; French Institute of Health and Medical Research (INSERM), Paris, France; École Pratique des Hautes Études, Montpellier, France

**Keywords:** Global activity limitation indicator, Health expectancy, Disability-free life expectancy, Healthy life years, Disability, Functioning, Measurement

## Abstract

**Background:**

The Global Activity Limitation Indicator (GALI), the measure underlying the European indicator Healthy Life Years (HLY), is widely used to compare population health across countries. However, the comparability of the item has been questioned. This study aims to further validate the GALI in the adult European population.

**Methods:**

Data from the European Health Interview Survey (EHIS), covering 14 European countries and 152,787 individuals, were used to explore how the GALI was associated with other measures of disability and whether the GALI was consistent or reflected different disability situations in different countries.

**Results:**

When considering each country separately or all combined, we found that the GALI was significantly associated with measures of activities of daily living, instrumental activity of daily living, and functional limitations (P < 0.001 in all cases). Associations were largest for activity of daily living and lowest though still high for functional limitations. For each measure, the magnitude of the association was similar across most countries. Overall, however, the GALI differed significantly between countries in terms of how it reflected each of the three disability measures (P < 0.001 in all cases). We suspect cross-country differences in the results may be due to variations in: the implementation of the EHIS, the perception of functioning and limitations, and the understanding of the GALI question.

**Conclusion:**

The study both confirms the relevance of this indicator to measure general activity limitations in the European population and the need for caution when comparing the level of the GALI from one country to another.

**Electronic supplementary material:**

The online version of this article (doi:10.1186/1471-2288-15-1) contains supplementary material, which is available to authorized users.

## Background

Health expectancies are increasingly used as summary measures of population health. Since 2005, the European Union (EU) has monitored an indicator of life expectancy without activity limitations, known as "Healthy Life Years" (HLY). Increasing HLY is crucial for the EU in order to reduce the social and economic burden associated with life expectancy lengthening. The European Innovation Partnership on Active and Healthy Ageing is targeting a two-year increase in HLY by 2020 [1, 2].

The European Commission deployed efforts to harmonise the data used in EU Member States to measure HLY, in contrast to other health expectancies data which often lack international harmonisation [3]. The Statistics of Income and Living Conditions (SILC) survey, coordinated by Eurostat, provides the data on activity limitations via the Global Activity Limitation Indicator (GALI). The GALI belongs to the family of disability indicators, targeting situations in which health disorders and conditions have impacted people’s usual activities. It is a single-item survey instrument which was developed with an explicit definition of the underlying concepts, which facilitates its translation and inclusion into different European surveys [4]. Since the inclusion of the indicator in the SILC, there have been efforts to improve its harmonisation, including a major revision of the translations into EU languages in 2008.

Despite harmonisation in data collection, cultural understanding and differences in reporting may threaten (cross-national) validity of the GALI. In Belgium, the GALI performed appropriately against other health indicators [4]. In France, the GALI was also strongly associated with functional and activity limitations, but the association with these other measures varied by education level and employment status, independently of the level of general health [5, 6]. A first cross-national study [7] including 11 countries from the Survey of Health, Ageing and Retirement in Europe revealed consistent relationships across countries between the GALI and other measures of disability in the older population. Whether these results hold for the wider adult European population remains unknown.

Using data from the European Health Interview Survey (EHIS), we assessed how the GALI is associated with other measures of disability, and whether the GALI is consistent or reflects disability differently among adults in 14 European countries. Our study, which is part of the EU Joint Action on Healthy Life Years [8], contributes to a better understanding of the European indicator HLY in providing a first cross-validation of the GALI in adults aged 15 years and older.

## Methods

### Data

The EHIS was conducted between 2007 and 2010 in individuals aged 15 years and older in 19 countries. Relevant data from 14 countries was available at the time of the analysis: Belgium, Bulgaria, Cyprus, Czech Republic, France, Greece, Hungary, Latvia, Malta, Poland, Romania, Slovakia, Slovenia, and Spain (Table 1). Microdata were analysed at the premises of Eurostat. Procedures for application for access to the microdata are given by Eurostat ( http://ec.europa.eu/eurostat/web/microdata/overview).

**Table 1:** **Characteristics of the European Health Interview Survey population, by country** ^**a**^, 2007-2010

		Belgium	Bulgaria	Cyprus	Czech Republic	France	Greece	Hungary	Latvia	Malta	Poland	Romania	Slovakia	Slovenia	Spain
Data collection year		2008-9	2008	2008	2008	2008	2009	2009	2008	2008	2009	2008	2009	2007	2009-10
Sample size (N)		9,651	5,661	6,931	1,955	24,689	6,172	5,051	6,458	3,669	35,100	18,172	4,972	2,118	22,188
response rate (%)		60.0	73.8	81.6	56.0	-	95.5	80.6	72.0	72.0	72.0	89.0	66.0	-	74.0
Mean age (yrs)		47.1	46.6	43.3	44.4	46.3	47.3	47.3	45.3	46.8	44.6	44.4	43.3	46.0	46.5
% Male		48.1	47.9	49.2	48.6	47.8	48.8	46.6	45.3	46.4	47.6	48.3	48.1	49.1	49.0
GALI	% limited	20.8	24.1	18.1	27.8	25.2	22.8	41.0	43.6	24.6	24.6	22.5	38.2	36.3	24.4
ADL	% having difficulty with 1 or more activity	10.1	10.3	5.3	10.1	3.4	6.5	14.3	12.1	5.8	9.4	5.9	9.6	15.7	7.3
IADL	% having difficulty with 1 or more activity	-	23.1	13.0	14.2	-	18.2	18.5	23.2	9.7	18.4	12.3	13.7	18.3	14.3
Functioning % limited		26.2	28.2	20.3	21.6	27.6	28.6	32.7	33.8	27.2	21.4	28.6	27.3	30.3	26.9

*GALI* Global Activity Limitation Indicator, *ADL* activities of daily living, *IADL* instrumental activities of daily living.

^a^Results account for survey designs using sampling weights.

The sample sizes range from 1,955 (Czech Republic) to 35,100 (Poland) persons per country, representing the adult population (including institutional population in Belgium, Czech Republic, France and Malta). Response rates varied between 56.0% (Czech Republic) and 95.5% (Greece). Proxy interviewing was used in most countries (varying from 0.7% (Greece) to 26.0% (Cyprus)) and participation was mandatory for Cyprus, France, Spain and Greece [9].

The EHIS questions were implemented either as one specific survey or as elements of existing national surveys, following Eurostat translation protocols from the original English questionnaire. Sampling design and data collection methods varied from country to country [9], but (normalised) sampling weights were available for the analyses. Examination of the question wording and responses categories revealed missing questions or comparability problems in some countries [10]. Non-comparable variables were considered as missing in these countries. For each respective analysis countries with missing variables were excluded along with the respondents who had missing values on the key variables.

### Measurements

#### Global Activity Limitation Indicator (GALI)

The Global Activity Limitation Indicator (GALI) is a single-item survey instrument reported by the individual him or herself to assess health-related activity limitations: "For at least the past six months, to what extent have you been limited because of a health problem in activities people usually do?". Possible responses are: severely limited, limited but not severely and not limited at all. The indicator refers to general restrictions in activity without specifying the type of activity concerned (work, household chores, leisure, personal care etc.). Because of the low numbers reporting themselves as severely limited, severe and moderate limitations were merged into one category (limited), as is commonly done when calculating the HLY indicator [11].

### Limitations in activity of daily living (ADL)

Difficulties in activities of daily living correspond to the most severe level of activity limitations [12]. The measure is based on the difficulty or need of assistance for basic activities everyone is expected to perform independently: washing, getting (un)dressed, feeding, getting in and out of bed,

using the toilet. Severe difficulties with these activities reflect situations of dependence requiring human assistance. These situations create social exclusion in many areas of life. ADL scores (‘any ADL’ and ‘sum of ADL’) were created using 5 activities of daily living questions, independently of the level of severity [13].

### Limitation in instrumental activity of daily living (IADL)

Difficulties in instrumental activities of daily living are broader than ADL limitations and concern domestic activities which allow an individual to live independently [14]: difficulty or need of assistance for preparing meals, using the telephone, shopping, managing medication, doing light housework, doing occasional heavy housework, taking care of finances and everyday administrative tasks. IADL scores (‘any IADL’ and ‘sum of IADL’) were created using 7 instrumental activities of daily living questions, independently of the level of severity [13]. A filter was applied to the IADL questions to account exclusively health-related limitations.

### Functional limitations

Functional limitations refer to difficulties in performing basic actions used in daily life [15]. Unlike ADL/IADL indicators, the functional limitations indicator evaluates the level of severity: severity of functional limitations - as opposed to the number of domains with limitations - impacts more directly the risk of meeting difficulties in activities. A summary variable measuring functioning by severity (no limitation; any moderate but no severe limitation; any severe limitation) was constructed from 6 items assessing the extent of limitation in physical actions: walking a certain distance, going up or down stairs, squatting and kneeling, carrying in the hands or in the arms, using hands and fingers to manipulate small objects, biting and chewing [16, 17]. A dichotomous variable (any limitation vs. none) was also constructed.

### General approach

To investigate the validity of the GALI, we compared it with three standard measures of disability. None of these instruments is a gold standard for the GALI, but positive associations with the GALI are expected. The GALI taps limitations in the performance of social roles and activities (such as work, school, parenting, leisure) and has a broader definition of activity than ADL and IADL measures [4, 16]. Association of IADL and ADL measures with the GALI should be strong and stronger for the ADL measure, which captures more disabling limitations than the IADL measure [7]. Most people reporting daily activity limitations should report global activity limitations; but, some individuals reporting global activity limitations might be limited in other activities than basic daily activities.

In the disablement process, functional limitations immediately precede activity limitations [15]. Since individuals with functional limitations have a higher risk of being limited in activities, we expect some individuals with functional limitations to report activity limitations. However, not all individuals with functional limitations have long-term limitations in their activities, so the association between the GALI and functional limitations could be weaker than with daily activity limitations.

In addition to examining patterns in the associations across all countries, this study further evaluates to what extent the magnitude of these associations varies between countries.

### Statistical methods

The relationships between the GALI and three measures of disability was investigated 1) in the European population as a whole, and 2) in every country separately, which allowed us to test whether the relationships were consistent in different countries. We performed additional analyses, restricted to those aged 50 years and over, for comparison with those reported by Jagger et al. using the Survey of Health, Ageing and Retirement in Europe (SHARE) [7].

1. Pooled analyses: for every measure used to evaluate the GALI (sum of ADL, sum of IADL and functional limitations by severity; all treated as categorical variables), a logistic regression model was fitted, adjusting for the effects of gender, age (measured in years) and interactions between the predictors, if statistically significant (α = 0.05). The probability of being classed as limited or not limited for the GALI was estimated for each category or value of the measure of interest. The relationship between the GALI and each measure was assessed.
2. Country-specific analyses: treating each national EHIS sample separately, logistic regression models were fitted to estimate the odds ratios between the GALI and each disability measure (dichotomised as no limitation vs. at least one limitation), adjusting for age and gender. We used dichotomised versions of the disability measures to avoid sparseness and to ease comparability with the SHARE study. Random-effect meta-analysis models were then fitted using country-specific odds ratios from the logistic regression models (odds ratios >100 were excluded). We assessed heterogeneity between countries in the association between the GALI and the three other measures with Cochran’s Q test and Higgins I^2^ statistic [7, 18, 19].

## Results

The 14 countries were fairly similar in terms of age and gender compositions, with a mean age of between 43.3 years (Cyprus and Slovakia) and 47.3 years (Greece and Hungary) and a proportion of men varying from 45.3% (Latvia) to 49.2% (Cyprus) (Table 1).

The percentage reporting global activity limitations varied widely by country and was lowest in Cyprus (18.1%) and highest in Latvia (43.6%). When compared to the SILC 2009, these figures were higher in 7 countries (i.e. more than 5% differences), as illustrate age-standardised and gender comparisons (Additional file 1).

The distribution of limitations measured by the other measures of disability also showed substantial variations by country. The percentage with at least one ADL limitation was lowest in France (3.4%) and highest in Slovenia (15.7%); the percentage with at least one IADL limitation was lowest in Malta (9.7%) and highest in Latvia (23.2%); and the percentage with at least one functional limitation was lowest in Cyprus (20.3%) and highest in Latvia (33.8%). The magnitude of limitations converged across the four measures in some countries: Hungary, Latvia and Slovenia displayed high levels of disability on the four measures; whilst Cyprus had among the lowest prevalence levels.

We then examined the global association between the GALI and other disability measures. Of people reporting ADL and IADL limitations, 89% and 82% respectively were likely to report global activity limitations (Table 2). The probability to report global activity limitations among those who have neither ADL nor IADL limitations was about 20%. A third of people with functional limitations did not consider themselves as limited in their activities. People who reported none of the functional limitations listed in the questionnaire had a very low probability of reporting global activity limitations.

**Figure 1:**
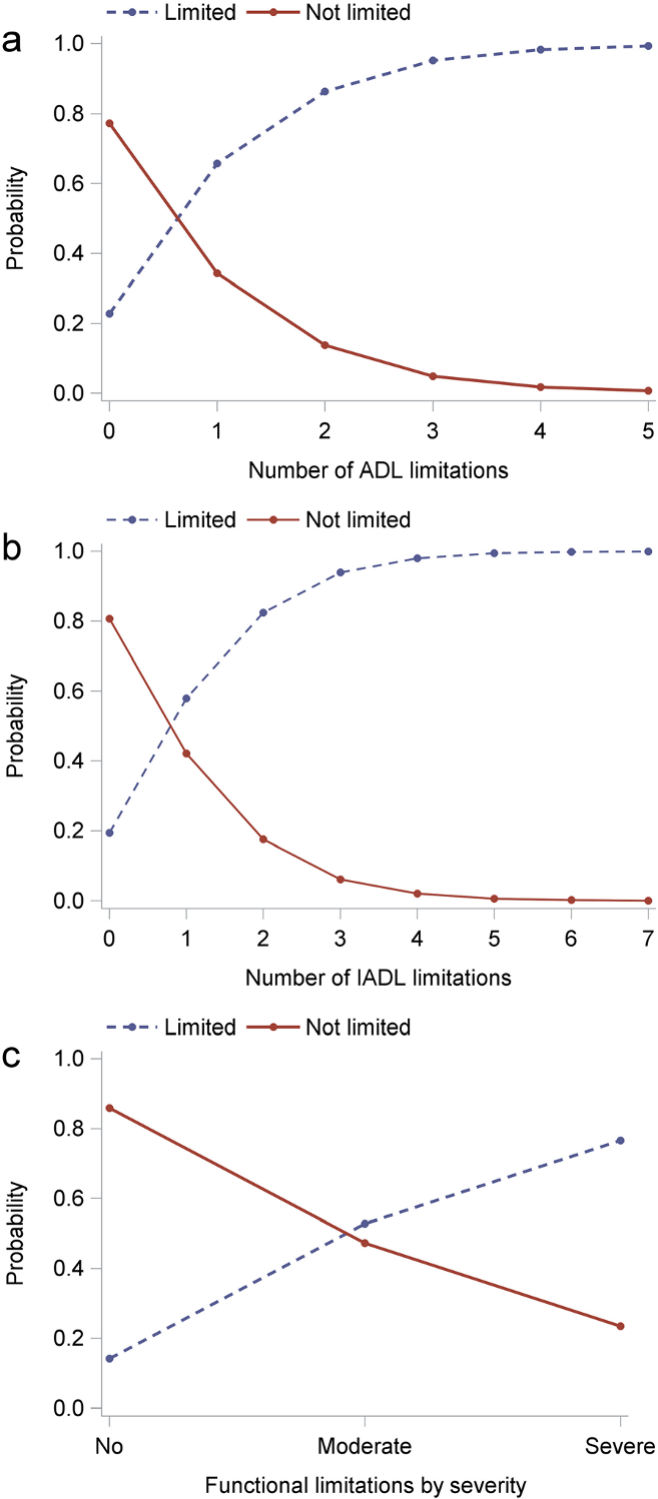
**Probability distribution of the GALI against other measures of disability, European Health Interview Survey, 2007-2010. (a)** activities of daily living (ADL) limitations; **(b)** instrumental activities of daily living (IADL) limitations; **(c)** physical functional limitations. No comparable data were available for Belgium and France in **(b)**.

The probability of reporting limitation based on the GALI increased as the number of ADL and IADL limitations increased (up to about 99% for respectively 5 and 7 ADL/IADL limitations) (Figure 1). The association was less pronounced with functional limitations by severity (no functional limitation: 14%; at least one severe functional limitation: 76%).

**Figure 2:**
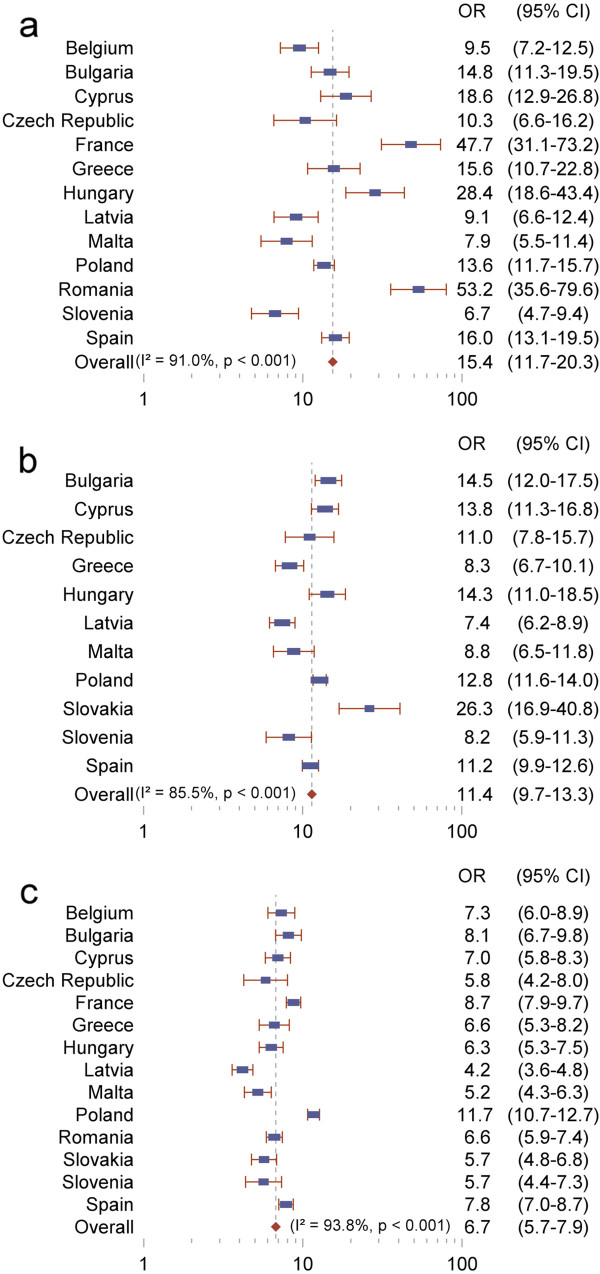
**Cross-country comparison of association between the GALI and other measures of disability, European Health Interview Survey, 2007-2010. (a)** activity of daily living (ADL) limitations; **(b)** instrumental activities of daily living (IADL) limitations; **(c)** physical functional limitations. Weights are from random effects analysis. Slovakia **(a)** and Romania **(b)** were excluded because of extreme values (odds ratio (OR) > 100) and no comparable data were available for Belgium and France in **(b)**. Source: European Health Interview Survey.

Next, we determined whether the GALI measured disability similarly across countries, that is, whether individuals identified as having disability, defined using ADL, IADL or functional limitations, had similar probabilities of reporting GALI limitations between the different EU countries. Results from the meta-analyses revealed significant positive associations of the GALI with each measure (Figure 2). In all countries, respondents with disability were more likely to report limitations based on the GALI (P < 0.001 in all cases), indicating a consistency in the associations. The overall odds ratios estimated from the meta-analyses were largest for ADL (15.4), intermediate for IADL (11.4) and lowest though still high for functional limitations (6.7). The more selective the disability indicator, the stronger the association with the GALI: individuals with more severe types of disability are more prone to report global activity limitations than people with milder forms of disability.

**Table 2:** **Predicted probability of the GALI-defined activity limitations by other measures of disability, European Health Interview Survey, 2007-2010**

		GALI	
		Not limited	Limited
ADL limitations	No	0.78	0.22
	1+	0.11	0.89
IADL limitations	No	0.82	0.18
	1+	0.18	0.82
Functional limitations	No	0.86	0.14
	1+	0.34	0.66

*GALI* Global Activity Limitation Indicator, *ADL* activities of daily living, *IADL* instrumental activities of daily living.

Heterogeneity in the size of the odds ratios reached statistical significance in all analyses (P < 0.001; I^2^ > 50%): the relationship between the GALI and other measures was more pronounced in some countries compared with others. For instance, the odds of being limited by the GALI if an individual had difficulties with at least one ADL compared with none varied from 6.7 (95% CI: 4.7, 9.4) for Slovenia to 53.2 (95% CI: 35.6, 79.6) for Romania. Variability in the point estimates of the odds ratios was intermediate for IADL (odds ratios varying from 7.4 to 26.3) and lowest for functioning (odds ratios varying from 4.2 to 11.7; 85% of the odds ratios comprised between 5.2 and 8.7).

However, most countries showed consistent patterns of associations with the GALI for ADL and IADL analyses, but not for functioning: countries with a strong association between the GALI and ADL also tended to exhibit a strong association between the GALI and IADL. For instance, Bulgaria had odds ratios of 14.8 and 14.5 in ADL and IADL analyses respectively.

Overall, the size of the associations differed between countries, but the pattern within countries was the same: stronger associations between ADL and IADL and the GALI, weaker association with functional limitations.

## Discussion

When considering each country separately or all combined, we found that the GALI consistently appeared to be a good indicator of disability in the adult population, corroborating previous studies [4, 7]. Our study confirmed a stronger association between the GALI and ADL, intermediate with IADL and somewhat lower with functioning.

We further observed that the strength of the associations that relates the GALI to disability measures varied across countries. Further analyses (not reproduced here) using different subsets of countries (EU-15 countries; excluding outliers; or excluding countries with large sample sizes (Belgium, France, Poland, Romania, Spain)) reached similar conclusion. This result partly challenges a former study of the older European population, using the SHARE [7]. To understand differences in the results, we mimicked the SHARE study using the EHIS participants aged 50 years and older (see Additional file 2). Comparison with the SHARE was possible for ADL and IADL analyses although the questions slightly differed between the two surveys. Apart from some extreme odds ratios, results were very comparable. Countries included in both studies had comparable estimates (i.e. Belgium, Greece and Spain), with the exception of France which had an extreme value in our study due to lower ADL limitations compared to other countries. Yet, our study had more power and reached statistical significance for both ADL and IADL heterogeneity tests even after the exclusion of outliers, whereas the SHARE study found significant heterogeneity for IADL only.

It appears that the main difference with the SHARE study was that, in our study, ADL and IADL measures were extremely strong predictors of the GALI in some countries. Respondents of those countries tended to associate ADL and IADL measures to the GALI more often. For example, in Romania, 99.8% of the respondent who reported no IADL limitations, reported no limitation on the GALI. We did not find these high associations in other countries.

We suspect survey effects to be (partly) responsible for these differences. The SHARE survey methodology is centrally managed with no room for variability in the survey mode and method. As opposed to SHARE, the EHIS is not implemented homogeneously across countries, resulting in various administration modes, population and sampling frames (e.g. inclusion or exclusion of the institutionalised population), sampling designs, item non-responses rates, uses of proxies and even question orders [9]. These survey characteristics impact survey responses [20, 21] and are likely to hamper comparability if they change from country to country. For example, item non-response rates varied greatly across countries and may cause different selection biases. For IADL, the proportion of individuals analysed was highest in Cyprus (almost 100%) and lowest in Poland (87%).

Another explanation may come from the self-reported nature of the data. The GALI and the other measures of disability are subject to social and cultural variations in reporting, even in perfectly harmonised surveys. Variation in the GALI prevalence and in its association with other measures may therefore be influenced by reticence about reporting global activity limitations or other types of disability. This may explain why the SHARE study found no significant cross-country variability in the association between the GALI and the objective measures of disability (i.e. maximum grip strength and walking speed). As to the GALI itself, it particularly triggers reporting variations by referring to usual activities in the question: "have you been limited […] in activities people usually do". Depending on age, culture, social background and country, individuals may interpret limitations in usual activities differently, independently of their disability situation. In order to account for heterogeneity in reporting styles, recent statistical techniques using anchoring vignettes were developed [22]. For example, such vignettes enabled the estimation of cross-country differentials in work disability reporting [23]. Having such tools for the GALI question would certainly contribute to better understanding of the threshold used by respondents to report or not to report activity limitations.

Our study is the first comprehensive evaluation of the GALI in the adult population of 14 European countries. Its strengths are the number of countries covered in the EHIS survey and the data quality in terms of the range of measures of disability, the sample sizes and population coverage (i.e. the whole adult population). A limitation is the differential survey methods across the EHIS participating countries.

As we validated the GALI using measures which are also self-reported, we could not, in contrast to the SHARE study [7], demonstrate that the GALI reflects objectively measured activity limitations. What we showed, however, is that the GALI performs well for reporting perceived activity limitations. We confirmed that the GALI is a useful global instrument for measuring activity limitations in the fashion self-rated health assesses general health status.

The GALI - as the measure underlying the European indicator HLY - should be validated in the adult European population. The fact that, in most countries, we observed consistent and gradual associations with the GALI for ADL, IADL and functioning is encouraging and suggests that the GALI question is understood similarly in different countries. Yet, a few countries stood out by extreme results. We suspect that the lack of harmonisation of the EHIS is responsible for these inconsistencies. That is to say, we may be facing an issue of survey data comparability rather than one of cross-national validity of the GALI itself [24].

Whether this explanation holds or not, our results have implications for the indicator HLY when based on the EHIS. HLY based on the SILC - which is the data source for the indicator - may suffer from similar harmonisation problems at the level of the implementation of the survey [25]. Similar outlying countries are therefore likely to exist in the SILC data, which may in turn influence the HLY figures and hamper their comparability. We believe that improving further the harmonisation of the data collection of the EHIS and the SILC across the European countries is necessary to enhance the quality of the HLY figures across Europe. Further understanding of the methodological, cultural and health factors influencing the GALI is needed in order to fully validate and compare HLY figures across countries.

## Conclusion

The study both confirms the relevance of the GALI to measure general activity limitation in the European population and highlights the need for caution when comparing the levels of the GALI from one country to another; analysis of patterns and trends should be preferred when looking at European disability and HLY.

## Electronic supplementary material

Additional file 1: **Age-standardised comparison of the GALI distribution (% limited) between the EHIS and the SILC 2009.** Table comparing the GALI distribution by gender between the EHIS (European Health Interview Survey) and the SILC (Statistics on Income and Living Conditions) in 14 European countries. Results are age-standardised. (DOCX 16 KB)

Additional file 2: **Cross-country comparison of association between the GALI and other measures of disability in adults aged 50 years and older.** Figure describing the results of random-effect meta-analysis models to estimate the odds ratios between the GALI and (a) activity of daily living (ADL) limitations; (b) instrumental activities of daily living (IADL) limitations in adults aged 50 years and older. Slovakia (a) and Romania (b) were excluded because of extreme values (Odds Ratio (OR) > 100) and no comparable data was available for Belgium and France in (b). Source: European Health Interview Survey. (DOCX 268 KB)
