# Supplementary material for: Assessing the validity of the Global Activity Limitation Indicator in fourteen European countries
Source: BMC Med Res Methodol. 2015 Jan 2;15:1. doi: 10.1186/1471-2288-15-1 (PMC4298058; doi:10.1186/1471-2288-15-1)
Supplement: Supplementary file 1 — Additional file 1: Age-standardised comparison of the GALI distribution (% limited) between the EHIS and the SILC 2009. Table comparing the GALI distribution by gender between the EHIS (European Health Interview Survey) and the SILC (Statistics on Income and Living Conditions) in 14 European countries. Results are age-standardised. (DOCX 16 KB) [file 12874_2013_1155_MOESM1_ESM.docx]

Additional file 1. Age-standardised comparison of the GALI distribution (% limited) between the EHIS and the SILC 2009

|  | **Men** | | **Women** | |
| --- | --- | --- | --- | --- |
| **Country** | **SILC** | **EHIS** | **SILC** | **EHIS** |
| Belgium | 18.1 | 15.9 | 21.9 | 20.0 |
| Bulgaria | 13.0 | 18.3 | 14.1 | 22.4 |
| Cyprus | 16.2 | 16.1 | 17.2 | 19.4 |
| Czech Republic | 19.0 | 25.6 | 20.8 | 27.9 |
| France | 18.6 | 21.3 | 21.5 | 23.6 |
| Greece | 12.6 | 15.7 | 14.4 | 21.9 |
| Hungary | 24.3 | 34.5 | 25.3 | 38.0 |
| Latvia | 27.3 | 39.7 | 28.0 | 42.1 |
| Malta | 10.3 | 19.4 | 10.9 | 23.9 |
| Poland | 20.7 | 22.3 | 20.3 | 23.8 |
| Romania | 16.8 | 18.6 | 20.1 | 23.5 |
| Slovakia | 30.3 | 35.7 | 33.0 | 40.5 |
| Slovenia | 22.0 | 30.6 | 25.1 | 36.8 |
| Spain | 19.8 | 18.6 | 23.4 | 24.3 |

Table comparing the GALI distribution by gender between the EHIS (European Health Interview Survey) and the SILC (Statistics on Income and Living Conditions) in 14 European countries. Results are age-standardised.
