# Supplementary material for: Assessing the validity of the Global Activity Limitation Indicator in fourteen European countries
Source: BMC Med Res Methodol. 2015 Jan 2;15:1. doi: 10.1186/1471-2288-15-1 (PMC4298058; doi:10.1186/1471-2288-15-1)
Supplement: Supplementary file 2 — Additional file 2: Cross-country comparison of association between the GALI and other measures of disability in adults aged 50 years and older. Figure describing the results of random-effect meta-analysis models to estimate the odds ratios between the GALI and (a) activity of daily living (ADL) limitations; (b) instrumental activities of daily living (IADL) limitations in adults aged 50 years and older. Slovakia (a) and Romania (b) were excluded because of extreme values (Odds Ratio (OR) > 100) and no comparable data was available for Belgium and France in (b). Source: European Health Interview Survey. (DOCX 268 KB) [file 12874_2013_1155_MOESM2_ESM.docx]

**Additional file 2. Cross-country comparison of association between the GALI and other measures of disability in adults aged 50 years and older.**
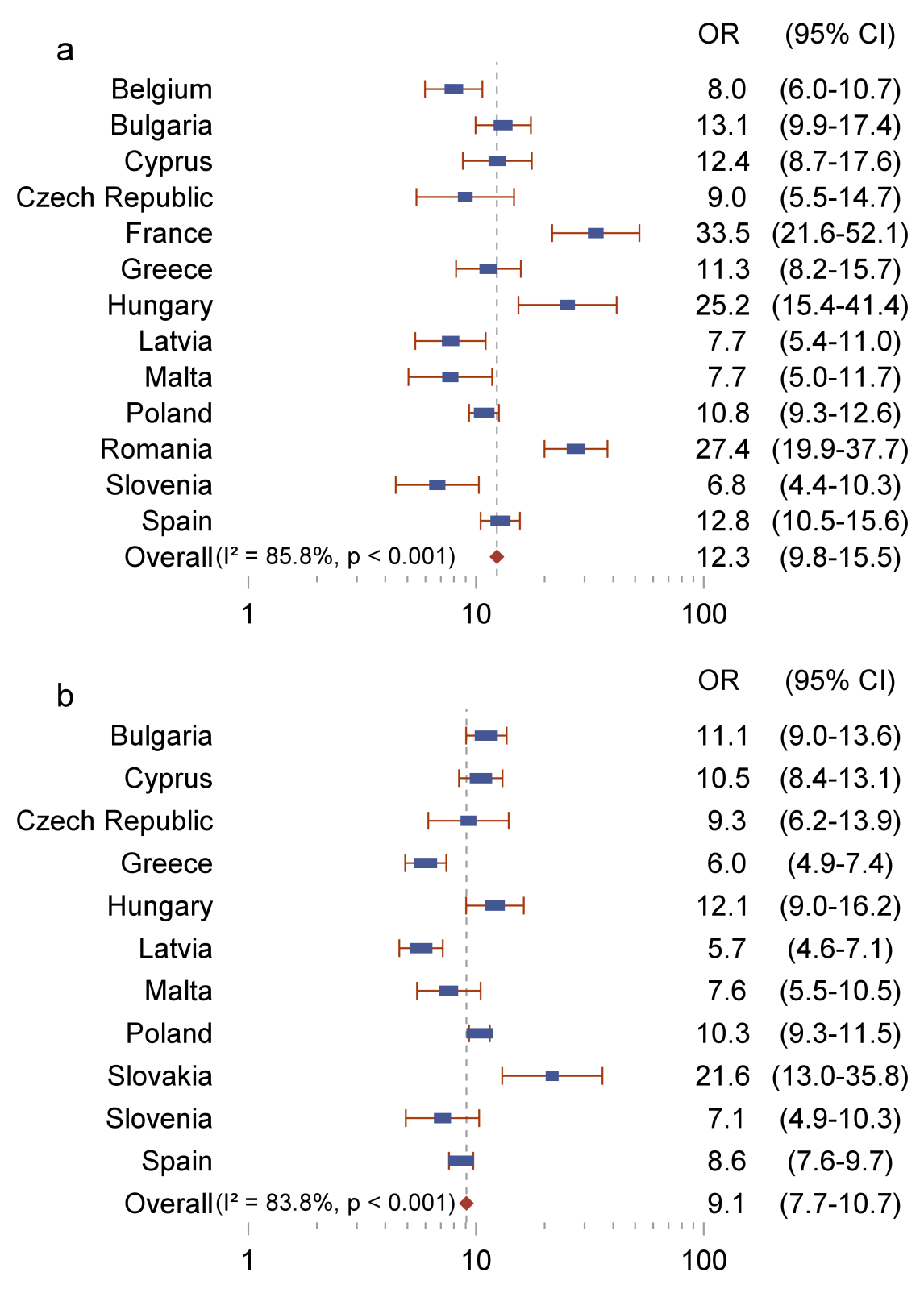


Figure describing the results of random-effect meta-analysis models to estimate the odds ratios between the GALI and (a) activity of daily living (ADL) limitations; (b) instrumental activities of daily living (IADL) limitations in adults aged 50 years and older. Slovakia (a) and Romania (b) were excluded because of extreme values (Odds Ratio (OR) > 100) and no comparable data was available for Belgium and France in (b). Source: European Health Interview Survey.
